# Accelerometer measured physical activity patterns of children during segmented school day in Abu Dhabi

**DOI:** 10.1186/s12887-021-02639-7

**Published:** 2021-04-17

**Authors:** Rahma Ajja, Ludmilla F. Wikkeling-Scott, Keith Brazendale, Rafiq Hijazi, Abdishakur Abdulle

**Affiliations:** 1 Department of Health Sciences, College of Natural and Health Sciences, Zayed University, P.O. Box 144534, Abu Dhabi, United Arab Emirates; 2 Department of Public Health, All Saints University College of Medicine, Arnos Vale, Saint Vincent and the Grenadines; 3 Department of Health Sciences, College of Health Professions and Sciences, University of Central Florida, Florida, USA; 4 Department of Mathematics and Statistics, College of Natural and Health Sciences, Zayed University, Dubai, United Arab Emirates; 5 Public Health Research Center, New York University, Abu Dhabi, United Arab Emirates

**Keywords:** School children, Accelerometer, Moderate to vigorous physical activity

## Abstract

**Background:**

The overwhelming majority of United Arab Emirates (UAE) school-aged children do not meet the 60 min per day recommendation of moderate-to-vigorous physical activity (MVPA). Understanding the different school day segments contribution to children reaching this goal is a crucial step toward developing successful strategies to promote school day MVPA levels. This study aimed to objectively measure children’s’ physical activity levels and to examine the difference in physical activity levels between high active and low active children during the school day in Abu Dhabi.

**Methods:**

A total of 133 school children (56% boys; mean age 10.5 years; grades 4–7) from two elementary schools in Abu Dhabi wore accelerometers (ActiGraph GT9X Link) for up to 5 non-consecutive days during spring 2019. Children’s’ MVPA was estimated during the following school segments: class time, lunch, recess, physical education (PE), and whole school day. Children were stratified as “high active” meeting the ≥30 min/day MVPA school time guideline or “low active” accumulating < 30 min/day MVPA.

**Results:**

On average children accumulated 21.8 ± 22.6 min/day of MVPA on PE days and 22.4 ± 15.9 min/day of MVPA on non-PE days. Only 19% of children met the 30 min or more of school day MVPA recommendation, with higher proportion of boys (27%) meeting such recommendation compared to girls (8%). High active boys, spent the highest percent of time in MVPA during PE (28%), followed closely by lunch (27%). In compression, high active girls spent the highest percent of time in MVPA during lunch (14.2%) followed by recess (9.1%). High active children accumulated 15.06 more minutes of MVPA during PE (*p* < 0.001*)*, 2 more minutes during recess (*p* < 0.001*)*, 3 more minutes of MVPA during lunch (*p* < 0.001*)* and 5 more minutes of MVPA during class time (*p* < 0.001*)*.

**Conclusion:**

The overwhelming majority of school children did not meet the recommended 30 min /day MVPA during school time. Girls substantially accumulated less MVPA and more sedentary minutes across all segments during the school days compared to boys. Further research is needed to investigate school day segments contribution to children MVPA in the UAE.

## Background

Obesity among children and youth is a serious worldwide public health issue as current global trends indicate [1]. The United Arab Emirates (UAE) is no different, with reports showing 38–41% of UAE school aged children (13–17 years old) overweight, and 17–24% obese [2, 3]. Current literature indicates that one of the major causes of the childhood obesity is the “energy gap” defined as “imbalance between calories consumed and calories required” [4]. Physical activity is an essential part of the energy equation with low levels of daily physical activity seen as one of the key drivers of the global childhood obesity epidemic [5]. More specifically, a number of studies in the Gulf Country Council (GCC) region have linked school aged children Body mass index (BMI) and waist circumference with physical activity levels [6, 7]. Furthermore, time spent in sedentary behavior defined, as waking behavior characterized by energy expenditure ≤1.5 metabolic equivalence (METs) has also been identified as an independent health risk factor in children [8, 9].

Despite mounting evidence indicating the positive health benefits of participating in physical activity during childhood [10], the vast majority of children and youth in the UAE (both expatriate and UAE nationals) fall short of achieving the recommended amount of ≥60 min per day of moderate-to-vigorous intensity physical activity (MVPA) [3, 11]. Similar findings reported in other studies indicate that approximately 15% of girls and 25% of boys aged 13–15 years in seven Arab countries (including the UAE) engage in the recommended amount of daily physical activity [12]. More recently, reviews based on the UAE’s 2018 Report Card on physical activity for children and youth, reported an estimated 16% of the UAE children achieving the daily physical activity recommendation of ≥60 min of MVPA (M 21%; F 11%) across age groups and genders [13]. To add to current concerns, according to the World Health Organization the percentage of youth achieving daily physical activity recommendations declined from 27.5 to 16.7% between the year 2010 and 2016 in the UAE [3].

Over the past decade, the UAE government has made considerable efforts in confronting the issue of physical inactivity among the UAE population with a particular emphasis on youth in the school setting. Initiatives such as mandating that all schools offer 150 min of physical education (PE) per week in Dubai, the introduction of new physical and health education curriculum, and the “Eat Right And Get Active” program in the Abu Dhabi region schools are examples of the UAE Governments commitment to making the school environment more supportive of physical activity and healthy eating behaviors [14–16]. Currently there are no published physical activity recommendations for the amount of MVPA children should accumulate during the school day in the UAE, however, several other foreign national organizations recommend that children should attain at least 30 min of MVPA during regular school hours [17, 18]. The 30 min MVPA during school day is seen as achievable goal as the majority of children spend at least 7 h per day at school, where there are multiple school day segments (PE, Recess, Lunch) that could provide children with physical activity opportunities. To date, available data on UAE school-aged children’s physical activity levels and sedentary behaviors have been obtained from survey questionnaires [3, 11, 12], lacking objective measurements of these behaviors in the school setting. Thus, the purpose of this study was to objectively measure children’s’ physical activity levels and to examine the difference in physical activity levels between high active and low active children during the school day in Abu Dhabi.

## Method

### Study design and setting

This cross-sectional study used convenience sampling technique to recruit students (grades 4–7) attending two private schools in Abu Dhabi who expressed in interest during fall of 2018. Using the sample size formula for estimating a single population mean [19] with 95% confidence level (zα/2 = 1.96), margin of error (3 min) and standard deviation of approximately 20 min [20], a sample size of 171 children was required. The study sample size (167) is close to the required sample size obtained using the above formula. Data collection occurred in the spring of 2019 (January to April). Both schools were co-education settings (both genders). School 1 provided educational classes from foundation stage kindergarten (KG) through year 7 for around 900 students split across year groups of 2 to 5 classes each. School 2 had an enrollment of approximately 1600 students from KG through year 12 with the split across year groups of 2 to 5 classes each. In both schools, from KG to year 5 classes are mixed gender with separation of boys and girls starting in year 6. Both schools had covered outdoor spaces and large indoor space for PE classes. PE classes were scheduled for 50 min twice per week in both schools. For the year group of two classes, one class was randomly selected and all the children attending that class were invited to participate in this study. For the year group of more than 2 classes, at least 2 classes were randomly selected and children in those classes were invited to participate in the study. Classes were randomly selected by inputting the class identifier in to a random number generator. Parental written consent and child verbal assent were obtained for 167 child prior to data collection. Inclusion criteria for this study were children in grade 4–7, with exclusion criterion for participation in the study being inability to engage in physical activity without an assistive device such as the use of wheelchair or crutches.

### Anthropometric measures

Height was measured using a portable stadiometer (Seca 214 Portable Height Rod, Hamburg, Germany) to the nearest 0.1 cm and weight was measured using a digital scale (Model HD314; Tanita Corporation, Tokyo, Japan) to the nearest 0.1 Kg. All anthropometric measurements were recorded by the first and second authors. Children’s weights were classified according to the body mass index (BMI) percentile charts for age and sex from the Centers for Disease Control and Prevention (CDC) [19]. Healthy weight (<85th percentile), overweight (≥85th and < 95th percentile), and obese (≥95th percentile) [21]. Age (completed years), gender, and nationality were obtained from school records. All procedures were approved by Zayed University Institutional Review Board and by the Department of Education and Knowledge in Abu Dhabi (ADEK).

### Accelerometer data protocol

Children’s physical activity data were collected during school hours from 7:00 am to 2:00 pm using ActiGraph GT9X Link (Shalimar, FL) on a maximum of 5 non-consecutive school days (Sunday – Thursday) within a 4-month period (January to April) during Spring 2019. Accelerometer epoch was set at 5-s intervals to account for the sporadic nature of children’s physical activity [22]. During data measurement days, upon arrival to the school, participating children were fitted with a numbered accelerometer and the arrival time recoded (start time) by trained class teacher. After attaching the devices on the participating child’s hip (on the right side). Children were asked to wear the monitors while participating in all scheduled activities. At the end of the school day, trained class teachers collected the accelerometer devices from children at the family pickup/school bus departure area, and the time of departure was recorded (Stop time). Commonly-used accelerometer cut-point thresholds for MVPA intensity levels [23] and sedentary behavior [24] were used to distill the data. A valid school wear time was defined as having 4 or more hours of wear during school time (school time was determined using first bell and last bell for individual school) [25]. Non-wear time was defined as a period of 30 min of consecutive zeros and was removed from analyses [26]. Students with at least 1 day of valid accelerometer wear time were included in the finally analysis.

### Data analysis

All statistical analysis was performed using STATA version 12 (StataCorp, College Station, TX, 77845). Descriptive statistics including the frequencies and percentages were used to summarize the categorical data while the means and standard deviations were presented for continuous data. Independent two-sample t-tests were conducted to examine the differences in MVPA based on gender (Male, Female), physical activity status (‘high active’ ≥ 30 min/day MVPA, “Low active” < 30 min/day MVPA), and days with a PE session (Yes (1), No (0)). Chi-square test of independence was used to test the relationship between physically activity levels and gender. Multilevel mixed-effects linear regression was conducted, accounting for the nested nature of this data (e.g., school children in their classes were defined as first level and classes second level). The multilevel models were developed to identify the effect of child-level factors such as gender, age, nationality, weight status, and physical activity status on children’s accumulated MVPA for four discrete school day segments (class time, lunchtime, recess and PE period). Consistent with previous research, school day segments were identified based on school schedules [27].

## Results

Descriptive characteristics of the study sample are presented in Table 1. A total of 133 children (59 girls and 74 boys) out of 167 with at least 1 day of valid accelerometer data were included in the analysis. A total of 126 (95%) children had at least 4 days of valid accelerometer data. The mean age (±SD) of participants was 10.5 ± 1.1 years. The majority of the children were attending grades 6 & 7 (63%). More than half of study participants were Arab nationals (83%) and most of the study sample (53%) was of overweight/obese BMI category.

**Table 1:** Characteristics of study participants by gender

Variable	Total (*N* = 133)	Girls (*n* = 59)	Boys (*n* = 74)
Age ± SD^a^	10.5 ± 1.1	10.3 ± 0.8	10.7 ± 1.3
Grade			
4 and 5	49 (37%)	21 (36%)	28 (38%)
6 and 7	84 (63%)	38 (64%)	46 (62%)
Nationality			
Arab	111 (83%)	51 (86%)	60 (81%)
Non-Arab	22 (17%)	8 (14%)	14 (19%)
Height ± SD (cm)	144.7 ± 8.5	144.6 ± 8.0	144.8 ± 8.9
Weight ± SD (kg)	42.9 ± 10.9	42.7 ± 9.6	43.1 ± 11.9
BMI (kg/m^2^)			
Normal	62 (47%)	27 (46%)	35 (47%)
Overweight	49 (37%)	21 (36%)	28 (38%)
Obese	22 (16%)	11 (18%)	11 (15%)

^a^*SD* Standard deviation

Table 2 shows the proportion of children meeting 30 min of MVPA and the average time spent in different physical activity intensities and sedentary behavior during days when PE was and was not offered. The majority of children (81.2%) fail to meet 30 min MVPA when attending school, with a higher proportion of boys (27%) compared to girls (8%), achieving this threshold. On PE days, boys spent less time in sedentary activity (239 ± 74.4 min/day) and significantly more time in MVPA (26.6 ± 28.1 min/day), compared to girls who spent 263.1 ± 54.3 min/day in sedentary, and 15.9 ± 10.6 in MVPA activity respectively (*p* < 0.05). During the none-PE days boys accumulated significantly less sedentary minutes/day compared to girls (224.5 ± 48.4 and 257.7 ± 42.1 respectively) and significantly more MVPA minutes/day (25.9 ± 18.9) compared to girls (18.7 ± 10.6).

**Table 2:** Physical activity and sedentary minutes accumulated by gender

Variable	Total	Girls	Boys	*P*-value
	Mean (±SD)	Mean (±SD)	Mean (±SD)	
Physical activity level (Days with PE)				
Sedentary	250.2 (66.9)	263.1 (54.3)	239.7 (74.4)	0.048
LPA	87 (44.3)	91.2 (38.2)	83.5 (48.7)	0.318
MPA	13.7 (12)	11.8 (7.7)	15.3 (14.5)	0.081
VPA	8.1 (12.1)	4.1 (4.1)	11.3 (15.2)	0.000 *
MVPA	21.8 (22.6)	15.9 (10.6)	26.6 (28.1)	0.004*
Physical activity level (Days with no PE)				
Sedentary	240.3 (48.4)	257.7 (42.1)	224.5 (48.4)	0.000*
LPA	102.7 (32.5)	102.4 (33.7)	102.9 (31.4)	0.883
MPA	14.4 (8.5)	13.3 (7.3)	15.5 (9.4)	0.007*
VPA	8 (8.7)	5.4 (4.5)	10.4 (10.7)	0.000*
MVPA	22.4 (15.9)	18.7 (10.6)	25.9 (18.9)	0.000*
Active (≥ 30 min/day)	n(%)	n(%)	n(%)	
High	25 (18.8)	5 (8)	20 (27)	0.007*
Low	108 (81.2)	54 (92)	54 (73)	

Physical activity data presented in this table is based on a total of 596 valid days for 133 students with a total of 467 “No PE” days 129 days with PE; Significant difference* (*p* < 0.05)

*PE* Physical education, *LPA* Light physical activity, *MPA* Moderate physical activity, *VPA* Vigorous physical activity, *MVPA* Moderate to vigorous physical activity

**Fig. 1:**
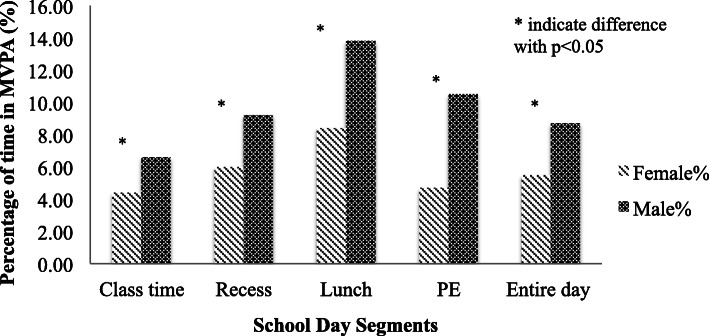
Proportion of time in moderate to vigorous physical activity

Figure 1 shows that. Overall, boys spent significantly higher proportion (*p* < 0.05) in MVPA across all school day segments in comparison to girls. Boys and girls spent the most parentage of time engaged in MVPA during lunch segment (13.8 and 8.4% respectively). Boys spent 10.5% of their PE time engaged MVPA where as girls spent 4.5% of their PE time engaged in MVPA (*P* < 0.05).

Table 3 shows high active and low active for each gender across different school day segments. The largest differences in the amount of MVPA accumulated between high active and low active boys and girls were during PE (22 and 3.5 min difference for boys and girls respectively) and class time (5.8 and 5.3 min difference for boys and girls respectively). The boys in the high active group spent the highest percentage of their time in MVPA during PE, whereas, the girls in the high active group spent the highest percentage of their time in MVPA during lunch. The boys and girls in the low active groups spent the highest percentage of their time in MVPA during lunch. The largest difference in the percentage of time boys spent in MVPA was during PE (23.2 difference in percent points), followed by lunch (18.2 difference in percent points). For girls the largest difference in percent of time spent in MVPA was during lunch (6.4 difference in percent points) followed by class time (5.0 difference in percent points). For boys, the smallest difference in percent time spent in MVPA was during class time for boys (7.0 differences in percent points), and PE for girls (1.8 difference in percent points).

**Table 3:** Comparison of high active and low active children across different school day segments

	Boys						Girls					
	MVPA Minutes			MVPA % of total time			MVPA Minutes			MVPA % of total time		
	High Active^a^	Low Active^b^	*P*-value	High Active^a^	Low Active^b^	*P*-value	High Active^a^	Low Active^b^	*P*-value	High Active^a^	Low Active^b^	*P*-value
Class time	10.3	4.5	0.000*	11.7%	4.7%	0.000*	9.6	4.3	0.000*	9.0%	4.0%	0.000*
Recess	4.4	1.5	0.000*	17.7%	5.9%	0.000*	2.3	1.4	0.024*	9.1%	5.7%	0.024*
Lunch	6.7	2.2	0.000*	27.0%	8.8%	0.000*	3.9	2.1	0.001*	14.2%	7.8%	0.002*
PE	26.6	4.6	0.000*	28.1%	4.9%	0.000*	8.5	5.0	0.228	6.3%	4.5%	0.490
Entire Day	47.0	18.0	0000*	13.4%	5.1%	0.000*	34.7	16.6	0.000*	9.3%	4.4%	0.000*

Data presented in this table are raw unadjusted means. Significant difference* (*p* < 0.05)

*MVPA* Moderate to vigorous physical activity

^a^High active ≥30 min of MVPA

^b^Low active < 30 min of MVPA

Model derived estimates for the amount of time school aged children spent in MVPA are presented in Table 4. BMI scores were unrelated to the amount of MVPA minutes accumulated across the different school day segments. Boys accumulated more MVPA minutes during recess [0.94 min/day (i.e., 56 s/day) (*p = 0.001*)], lunch [1.39 min/day (i.e., 83 s/day) (*p = 0.000)*] and class time [1.36 min/day (i.e., 81 s/day) (*p = 0.001)*]. Across all school day segments with each year increase in age, school aged children accumulated additional minutes in MVPA. Specifically 3.44 (*p = 0.003) more* minutes/day during PE, 0.36 (*p = 0.03*) more minutes/day (i.e., 21.6 s/day) during recess, 0.49 (*p = 0.041)* more minutes/day (i.e., 29 s/day) during lunch, and 0.56 (*p = 0.02)* more minutes/day (i.e., 34 s/day) during class time. High Active children (≥ 30 min/day MVPA) accumulated 15.06 more minutes of MVPA during PE (*p = 0.00)*, 2 more minutes during recess (*p = 0.00)*, 3 more minutes of MVPA during lunch (*p = 0.00)* and 5 more minutes of MVPA during class time (*p = 0.00)*.

**Table 4:** Association among demographic, activity status (high active vs. low active) and MVPA minutes accumulated during school day

Demographic and activity	Class Time				Lunch				Recess				PE			
	Coef.	SE	z	*P*-value	Coef.	SE	z	*P*-value	Coef	SE	Z	*P*-value	Coef.	SE	Z	*P*-value
Gender																
Boys (Ref)	1.36	0.41	3.31	0.001*	1.39	0.39	3.53	0.00*	0.94	0.27	3.47	0.001*	1.18	1.95	0.61	0.544
Age	0.56	0.24	2.33	0.02*	0.49	0.24	2.04	0.041*	0.36	0.17	2.16	0.03*	3.44	1.14	3.02	0.003*
Nationality																
Arab (ref)	0.91	0.38	2.35	0.019*	0.32	0.36	0.89	0.375	0.12	0.24	0.52	0.605	2.20	1.87	1.17	0.241
BMI (kg/m^2^) Normal (ref)																
Overweight	−0.13	0.30	−0.43	0.668	0.19	0.28	0.68	0.494	0.34	0.19	1.83	0.068	2.22	1.44	1.54	0.123
Obese	0.31	0.38	0.81	0.418	−0.02	0.35	−0.05	0.963	− 0.16	0.24	− 0.7	0.487	−1.44	1.82	−0.79	0.428
Activity status																
High active (Ref)	5.45	0.37	14.63	0.00	3.12	0.34	9.06	0.00*	1.91	0.23	8.23	0.00*	15.06	1.84	8.19	00.00*

Significant difference* (*p* < 0.05)

*MVPA* Moderate to vigorous physical activity, *BMI* Body Mass Index

## Discussion

This is the first study to objectively assess physical activity levels among school-age children in the UAE during school time. A substantial proportion (81%) of boys and girls fall short of meeting the 30 min per day school time MVPA suggested by the current guidelines. This finding is consistent with previous accelerometer-derived research estimates reporting that majority of children fail to meet the 30 min recommendation of MVPA during school day [27, 28]. In addition, the average accumulated MVPA minutes/day among children in the study was similar to reported averages in previous studies using hip mounted accelerometers that showed children’s daily MVPA minutes during school day ranged between 19.0 ± 0.7 and 28.5 ± 0.56 [29–32].

School settings can potentially expose children to extended periods of uninterrupted sedentary time [33]. The negative impact of sedentary time on children health has gained increasing attention in recent years independent of amount of time spent in MVPA [8, 34]. Accumulated sedentary minutes per school day among children in our study ranged from 240 ± 48.4 to 250 ± 66.9 min/day during PE and non-PE days. This is relatively similar to school time sedentary minutes estimate reported in earlier studies both in the GCC region and internationally [27, 28, 30]. Although not surprising, the considerable amount of sedentary time accumulated by children during both PE and non-PE school days is a call for concern, and highlights the need for strategies aimed at supporting school and teachers to integrate physical activities breaks and activity transitions in traditional lesson plans to minimize the amount of uninterrupted sedentary time (sedentary bouts) during the none-PE school segments.

Girls accumulated on average higher sedentary minutes/day and less MVPA minutes/day compared with boys, with only 8% of the girls meeting the 30 min/day of MVPA during school time. This finding is consistent with those reported in similar studies [27, 28]. A notable finding of this study is that girls classified as ‘high active’ accumulated less MVPA minutes/day across all segments of the day (class time, PE, recess, and lunch) with most of their MVPA minutes acquired during class time in comparison to boys. High active boys on the other hand, accumulated on average most of their MVPA minutes during PE segment followed by classroom segment. Our findings are inline with other studies which have shown that children do accumulate a large proportion of their MVPA minutes during class time [27]. In this study, since classroom data was not collected, it was difficult to assess if and what types of classroom activity strategies were used, if any, by teachers that could influence children’s MVPA levels during class time segment.

PE sessions have long been expected not only to aid children in meeting their daily MVPA minutes, but to also provide equal opportunities to attain MVPA minutes to those attending. Surprisingly, children in our study spent approximately similar minutes of MVPA on PE and non-PE days (21.8 ± 22.6 PE and 22.4 ± 15.9, respectively). This finding is inconsistent with previous literature reporting high minutes of MVPA accumulated during PE days among school age children [27, 32]. Moreover, in this study boys and girls spent relatively small proportion of their PE time segments engaged in MVPA (10.5 and 4.7%). These estimates are lower compared to those reported in Cheval and colleagues study [35] that found boys and girls spent around 32.02 and 27.78% of their PE time respectively engaged in MVPA. Observed discrepancies could be attributed to differences in PE structure and curriculum between schools in the US and UAE.

Similar to other school based studies our study found large difference in the accumulated minutes of MVPA between high active and low active children [27, 36]. This is further supported with model estimates where high active children accumulated meaningful amount of MVPA during PE segments (15 min more MVPA/day) in compression to low active children. Several studies have shown gender difference in physical activity among children in school settings [27, 28], our study indicate minimal difference with boy accumulating 1.18 more minutes of MVPA in PE segment compared to girls. BMI status was not associated with accumulated minutes of MVPA across all school day segments in our study. This is in contrast to previous school time research findings where higher BMI status was associated with lower volume and intensity of physical activity [6]. The reported variability in children’s MVPA during PE in this study, coupled with the fact that most of the children in the study failed to meet school day MVPA recommended guidelines and spent meaningfully small proportion of PE time engaged in MVPA is of concern, and highlight the need to reassess PE class structure (e.g., appropriateness of activities offered for both boys and girls) and to implement strategies aimed at maximizing the PE time (e.g., LET US Play principles) in order to increase the amount of MVPA minutes/day accumulated by school age children during PE sessions [27, 37, 38].

### Strengths and limitations

There are a number of strengths in this study. This is the first study to use accelerometers to measure children’s physical activity levels during school day, therefore providing the first objective measure for this age group in the UAE. Furthermore, this is one of the first studies to measure children’s physical activity levels across different school day segments in the UAE. Last, but not least, our study showed high compliance rate on accelerometer valid wear-time among participants with 133 children from 165 children (80.7%) wearing the accelerometer for at least 4 or more hours during school time.

Several limitations should be noted when interpreting the findings. First, the number of schools and the sample size are relatively small, therefore, care should be taken when generalizing the findings to other private schools in the city of Abu Dhabi, as well as public schools which could follow different PE schedule. Future studies should target a larger sample size from both private and public schools in Abu Dhabi. Secondly, class schedules provided by the schools were used to identify the different school segments. Although this is consistent with how similar studies identified school day segments [27], there is a possibility that reported schedules and the actual schedule followed by teachers could differ. Thirdly, the use of non-consecutive accelerometer measurement days is another limitation that should be considered, as this method of data collection does not take into account between days compensatory changes in levels of physical activity and sedentary behavior. Finally, the lack of contextual information on the types of activities offered in the different school day segments, specifically during PE sessions, further limits study findings. Such information may shed some light on the differences observed in MVPA estimates between boys and girls. Future studies should combine the use of accelerometers with methods that yield contextual information such as activity diaries and direct observations during random unannounced visits to the school.

## Conclusion

In summary, this study showed that the majority of boys and girls are not meeting the 30 min/day MVPA school time recommendation guidelines, with girls substantially accumulating less MVPA and more sedentary minutes across all segments during the school days compared to boys. PE and recess periods should be considered as the potential segments to provide children with opportunities to accumulate more minutes of MVPA. The findings highlight the needs for larger studies on school day segments contribution to children’s overall objectively measured MVPA in the UAE. Moreover, future studies should consider strategies that maximize the amount of time children spend in MVPA in PE in elementary school.

## Data Availability

Dataset used in this study is available with the corresponding author and can be provide upon request.

## Abbreviations

ADEK: Department of Education and Knowledge in Abu Dhabi
BMI: Body mass index
CDC: Centers for Disease Control and Prevention
GCC: Gulf Country Council
KG: Kindergarten
LPA: Light physical activity
MET: Metabolic equivalents
MPA: Moderate physical activity
MVPA: Moderate-to-vigorous physical activity
PE: Physical education
SD: Standard deviation
UAE: United Arab Emirates
US: United State
VPA: Vigorous physical activity
